# CAR T-Cells in Multiple Myeloma: State of the Art and Future Directions

**DOI:** 10.3389/fonc.2020.01243

**Published:** 2020-07-28

**Authors:** Luis Gerardo Rodríguez-Lobato, Maya Ganzetti, Carlos Fernández de Larrea, Michael Hudecek, Hermann Einsele, Sophia Danhof

**Affiliations:** ^1^Division of Medicine II, University Hospital Würzburg, Würzburg, Germany; ^2^Amyloidosis and Multiple Myeloma Unit, Department of Hematology, Hospital Clínic of Barcelona, Institut d'Investigacions Biomèdiques August Pi i Sunyer (IDIBAPS), Barcelona, Spain; ^3^Unit of Hematology and Bone Marrow Transplantation, IRCCS San Raffaele Scientific Institute, Milan, Italy

**Keywords:** multiple myeloma, immunotherapy, chimeric antigen receptor, B-cell maturation antigen, T-cell, cytokine release syndrome

## Abstract

Despite recent therapeutic advances, the prognosis of multiple myeloma (MM) patients remains poor. Thus, new strategies to improve outcomes are imperative. Chimeric antigen receptor (CAR) T-cell therapy has changed the treatment landscape of B-cell malignancies, providing a potentially curative option for patients who are refractory to standard treatment. Long-term remissions achieved in patients with acute lymphoblastic leukemia and Non-Hodgkin Lymphoma encouraged its further development in MM. B-cell maturation antigen (BCMA)-targeted CAR T-cells have established outstanding results in heavily pre-treated patients. However, several other antigens such as SLAMF7 and CD44v6 are currently under investigation with promising results. Idecabtagene vicleucel is expected to be approved soon for clinical use. Unfortunately, relapses after CAR T-cell infusion have been reported. Hence, understanding the underlying mechanisms of resistance is essential to promote prevention strategies and to enhance CAR T-cell efficacy. In this review we provide an update of the most recent clinical and pre-clinical data and we elucidate both, the potential and the challenges of CAR T-cell therapy in the future.

## Introduction

Multiple myeloma (MM), an incurable malignancy in most of the cases, is characterized by an uncontrolled proliferation of clonal plasma-cells in the bone marrow that produces aberrant quantity of monoclonal immunoglobulins (Ig) and end-organ damage (hypercalcemia, renal insufficiency, anemia, and/or bone lytic lesions). MM is the second most commonly diagnosed hematologic malignancy accounting for ~1% of all cancer types with an incidence rate of 6–7 cases per 100,000 persons per year (1).

The spectrum of treatment modalities of this disease has expanded significantly, with the introduction of novel agents including proteasome inhibitors (PI), immunomodulatory drugs (IMiD), and monoclonal antibodies (mAb). Despite these considerable advances, most MM patients eventually relapse and become resistant to treatment (1). In this regard, novel immunotherapeutic approaches have been developed to harness the intrinsic immune system against malignant cells.

Chimeric antigen receptor (CAR) T-cell therapy has emerged as potent treatment strategy against B-cell neoplasms with impressive outcomes and manageable toxicity (2–4). In the late 80's and early 90's, two international groups started working on CAR (5, 6). Kuwana et al. constructed a chimeric receptor molecule joining the Ig-derived variable regions and T-cell receptor-derived constant regions (5). Eshhar et al. (6) designed the CAR as a tool to equip T-cells with potent and specific anti-tumor efficacy and to overcome the limitations of the MHC-restricted T-cell activation in cellular cancer therapy, because reduction or loss of MHC class I molecules is frequently observed during malignant transformation. In the last 2 decades, efforts in the field focused on improving CAR design in order to promote efficacy and *in vivo* persistence of CAR T-cells. First-generation CARs have been replaced by more potent second- and third-generation CARs. Since 2003, CD19-targeted second-generation CARs have been developed and subsequently tested in B-cell malignancies. The FDA approvals of two CD19 CAR T-cell products in 2017 were based on results obtained from two pivotal studies showing remarkable results in patients with acute lymphoblastic leukemia and certain types of large B-cell lymphomas (2, 4). In MM, CAR-T cell therapy is still in its infancy. First clinical studies investigated CAR T-cells directed against Lewis Y antigen (7), CD19 (8), CD138 (9), and free light chain (10) in patients with relapsed/refractory (RR) MM. However, most promising results have been reported for BCMA-targeted CAR T-cells. Tremendous enthusiasm has fueled considerable efforts to define the optimal target antigen for CAR T-cell therapy in MM. Here, we discuss the latest outcomes of the most important clinical trials and provide an overview of different strategies to overcome resistance mechanisms against CAR T-cell therapy in MM.

## CAR Construct

**Figure 1:**
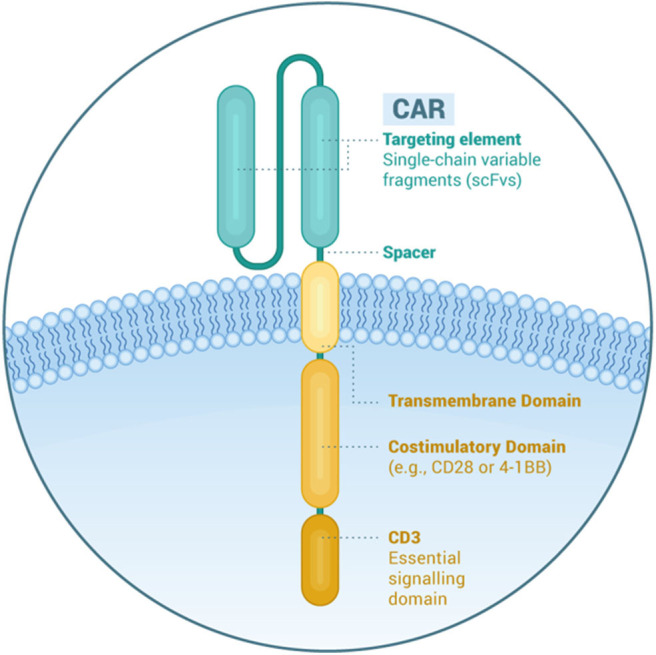
Structural elements of a chimeric antigen receptor.

A CAR is a recombinant receptor to re-direct T cells against selected antigens on the surface of tumor cells. It consists of different components (Figure 1). The extracellular binding moiety is usually derived from the heavy (VH) and light chain variable domains (LH) of a mAb that are linked in the form of single chain variable fragment (scFv). The hinge or spacer is designed with Ig-like domains, and the transmembrane domain from CD8α. The intracellular moiety contains the CD3ζ signaling chain of the T-cell receptor and provides the first signal for T-cell activation. Second and third generation CARs have one and two costimulatory domains, respectively (e.g., CD28, 4-1BB, or OX40) to promote CAR T-cell survival and proliferation. Fourth generation CAR T-cells, also known as armored CAR T-cells, produce cytokines that enhance CAR T-cell function or modify the tumor microenvironment (11).

## Target Antigens

The identification of suitable tumor-associated target antigens is essential for successful CAR T-cell therapy. In general, three prerequisites are required to enable both, effectiveness and safety. First, the antigen must be expressed on the tumor cell surface. Indeed, CAR binding occurs in an MHC-independent fashion (5) reducing the risk of immune escape due to HLA downregulation (12). However, expanding the pool of targetable antigens might allow the treatment of a wider spectrum of tumors, so TCR-mimetic CARs recognizing the tumor-antigen/HLA complex have been recently developed (13). Second, the antigen must be homogeneously expressed on the malignant cells and should ideally be essential for tumor survival (2). Finally, the target must be virtually absent from relevant healthy tissues to minimize on-target, off-tumor effects. Although no CAR T-cell therapy has been approved for the treatment of MM to date, several antigens are under investigation in early-phase clinical trials and preclinical studies (14).

## CAR Targets in Clinical Trials

### B-Cell Maturation Antigen

B-cell maturation antigen (BCMA; CD269, tumor necrosis factor receptor superfamily member 17/TNFRSF17) is a transmembrane glycoprotein and non-tyrosine kinase receptor. It shares similarities with two other receptors, which are B-cell Activating Factor of the TNF Family receptor (BAFF-R) and transmembrane activator, calcium modulator, and cyclophilin ligand interactor (TACI) (15–17). BCMA is expressed on the surface of late memory B-cells and plasma cells, and the expression is enhanced during B-cell differentiation. It is also expressed on plasmacytoid dendritic cells, but it is neither expressed on non-hemopoietic tissues, nor on hemopoietic stem cells, nor on naïve B-cells (15, 18–22). BCMA activation by its ligands, A Proliferation Inducing Ligand (APRIL) and B-cell Activating Factor (BAFF), transmits survival signals, and induces B-cell maturation and differentiation into plasma cells, as well as immunoglobulin (Ig) isotype switching (19, 23–25). In *in vivo* studies, BCMA knock-out mice display impaired survival of long-term plasma cells, but preserved B-cell development, Ig production and early humoral immune response (19, 26).

BCMA has been shown to play an important role in the pathogenesis of MM. It is expressed in MM cells and in human myeloma cell lines (27–33) and expression levels are higher on MM cells as compared to normal plasma cells (32, 34, 35). It is associated with proliferation, survival (by activation of AKT, MAPK, and NF-κB signaling cascades), and drug resistance by contributing to the immunosuppressive bone marrow microenvironment (27, 35, 36). Furthermore, it was reported that responses to donor lymphocyte infusions in MM patients after allogeneic stem cell transplantation were associated with the production of anti-BCMA antibodies, highlighting its role as immunotherapy target (37).

BCMA protein can be cleaved by gamma-secretase with subsequent release of soluble BCMA (sBCMA) (38). It is possible that sBCMA may also play a role in the pathogenesis of MM by sequestering circulating BAFF, thus preventing the stimulation of normal B-cells and plasma cells (39). In addition, high levels of sBCMA have been correlated with a worse prognosis in terms of progression-free survival (PFS) and overall survival (OS) and sBCMA was proposed as a novel biomarker to monitor and predict outcomes in MM (40–42).

The restricted expression of BCMA and its role in the pathogenesis of MM have made BCMA an attractive target for immunotherapies, especially CAR T-cells (21). Nevertheless, the role of the sBCMA remains to be elucidated, since it could interfere with BCMA-targeted immunotherapies (43). Several clinical trials with diverse BCMA-CAR T-cell constructs in patients with RRMM are ongoing, and the currently available data are encouraging. The next sections and Table 1 summarize the most important aspects of selected clinical trials with CAR T-cells targeting BCMA.

**Table 1:** Selected BCMA CAR T-cell clinical trials in multiple myeloma.

**Clinical trial/CAR**	**CAR construct specifications**	***N***	**Prior lines, median**	**Lymphodepletion protocol**	**CAR T-cell dose**	**Response**	**Survival**	**CRS (grade ≥ 3) CRS-related mortality ICANS (grade ≥ 3)**
NCI (44)	γ-retrovirus scFv murine CD28	24 (16 HD)	9.5 (HD)	Cy/Flu	0.3–9.0 × 10^6^/kg	ORR 81% (≥ CR 13%) MRD-neg 69% (HD)	mEFS 7.2 mo	CRS 94% (38%) CRS-RM 0% ICANS NA (19%)
Nanjing Legend/ LCAR-B38M (45, 46)	Lentivirus Bi-epitope VHH 4-1BB	57	3	Cy	0.07–2.1 × 10^6^/kg	ORR 88% (≥ CR 74%) MRD-neg 63%	mPFS 20 mo 18-mo OS 68%	CRS 89% (7%) CRS-RM 0% ICANS 2% (0%)
Nanjing Legend/ LCAR-B38M (47)	Lentivirus Bi-epitope VHH 4-1BB	17	4	Cy / Flu	0.21–1.52 × 10^6^/kg	ORR 88.2% (≥ CR 76.5%)	1-y PFS 52.9% 1-y OS 82.3%	CRS 100% (41.2%) CRS-RM 1 patient ICANS NA
CARTITUDE-1/ JNJ68284528 (LCAR- B38M) (48, 49)	Lentivirus Bi-epitope VHH 4-1BB	29	5	Cy/Flu	0.75 × 10^6^/kg	ORR 100% (sCR 86%) MRD-neg 100% (n=23)	9-m PFS 86%	CRS 93% (7%) CRS-RM 1 patient ICANS 10% (3%)
UPenn/CART- BCMA (50)	Lentivirus scFv human 4-1BB	25	7	Cohort 1: No Cohort 2: Cy Cohort 3: Cy	1: 1–5 × 10^8^ 2: 1–5 × 10^7^ 3: 1–5 × 10^8^	1: ORR 44% (≥ CR 9.1%) 2: ORR 20% (≥ CR 0%) 3: ORR 64% (≥ CR 11%)	1: mPFS 2.1 mo 2: mPFS 1.9 mo 3: mPFS 4.2 mo	CRS 88% (32%) CRS-RM 0% ICANS 32% (12%)
Bluebird bio/ CRB-401/bb2121/ idecabtagene vicleucel (51)	Lentivirus scFv murine 4-1BB	33	7–8	Cy/Flu	50–800 × 10^6^	ORR 85% (≥ CR 45%)	mPFS 11.8 mo	CRS 76% (6%) CRS-RM 0% ICANS 42% (3%)
KarMMA/bb2121/ idecabtagene vicleucel (52, 53)	Lentivirus scFv murine 4-1BB	128	6	Cy/Flu	150–450 × 10^6^	ORR 73% (≥ CR 33%)	mPFS 8.8 mo mOS 19.4 mo	CRS 84% (5.5%) CRS-RM 1 patient ICANS 18% (3%)
Bluebird bio/ CRB-402 /bb21217 (54)	Lentivirus scFv murine 4-1BB PI3K-inh	33	7	Cy/Flu	1: 150 × 10^6^ 2: 300 × 10^6^ 3: 450 × 10^6^	1: ORR 83% (≥ CR 33%) 2: ORR 43% (≥ CR 0%) 3: ORR 57% (≥ CR 14%)	NA	CRS 66% (6%) CRS-RM 1 patient ICANS 24% (8%)
Juno/EVOLVE/ JCARH125/ orvacabtagene autoleucel (55, 56)	Lentivirus scFv human 4-1BB EGFRt	44 62	7 6	Cy/Flu Cy/Flu	50–150 × 10^6^ 300–1,600 × 10^6^	ORR 82% (≥ CR 27%) ORR 92% (≥ CR 36%)	NA mPFS: 9.3 mo	CRS 80% (9%) CRS-RM 0% ICANS 25% (7%) CRS 89% (3%) CRS-RM 0% ICANS 13% (3%)
MSKCC MCARH171 (57)	Retrovirus scFv human 4-1BB EGFRt	11	6	Cy/Flu Cy	1: 72 × 10^6^ 2: 137 × 10^6^ 3: 475 × 10^6^ 4: 818 × 10^6^	ORR 64% (≥ CR 0%)	NA	CRS 60% (20%) CRS-RM 0% ICANS 10% (0%)
FHCRCFCARH143 (58)	Lentivirus scFv human 4-1BB EGFRt	7	8	Cy/Flu	1: 50 × 10^6^ 2: 150 × 10^6^ 3: 450 × 10^6^ 4: 800 × 10^6^	ORR 100% (≥ CR 36%)	NA	CRS 91% (0%) CRS-RM 0% ICANS 9% (0%)
Poseida TherapeuticsP-BCMA-101 (59, 60)	Non-viral PiggyBac scFv human 4-1BB	23	6	Cy/Flu	1: 0.75 × 10^6^/kg 2: 2 × 10^6^/kg 3: 6 × 10^6^/kg 4: 10 × 10^6^/kg 5: 15 × 10^6^/kg	ORR 43–100% at various doses	NA	CRS 9.5% (0%) CRS-RM 0% ICANS 4.8% (4.8%)
BRD015Tongji Hospital (61)	Lentivirus scFv murine CD28	28	NA	Cy/Flu	5.2–25 × 10^6^/kg	ORR 89% (≥ CR 61%)	BCMA ≥50%: 9.8 mo BCMA <50%: 2.1 mo	CRS NA (14.3%) CRS-RM 0% ICANS NA (NA)
CT053Multicenter China (62)	Lentivirus scFv human 4-1BB	24	4.5	Cy/Flu	1.5 × 10^8^/kg	ORR 87.5% (≥ CR 79%)	mPFS 9.2 mo	CRS 62.5% (0%) CRS-RM 0% ICANS 12.5% (4%)
CT103A (63)	Lentivirus scFv human 4-1BB	17	4	Cy/Flu	1–6 × 10^6^ /kg	ORR 100% (≥ CR 71%)	NA	CRS 94.4% (28%) CRS-RM 0% ICANS 0% (0%)
FHVH-BCMA (64, 65)	γ-retrovirus FHVH33 human 4-1BB	12	6	Cy/Flu	0.75–3 × 10^6^/kg	ORR 83.3% (≥ CR 16.7%)	NA	CRS 91.7% (8.3%) CRS-RM 0% ICANS 25% (8.3%)
HRAIN Biotechnology (66)	γ-retrovirus scFv murine 4-1BB EGFRt	20	5.5	Cy/Flu	9 × 10^6^/kg	ORR 79% (≥ CR 45%)	mPFS 15 mo	CRS 45% (5%) CRS-RM 0% ICANS NA (7%)

*CAR, chimeric antigen receptor; CR, complete response; CRS, cytokine-release syndrome; CRS-RM, CRS related-mortality; Cy, cyclophosphamide; EFS, event-free survival; EGFRt, truncated epidermal growth factor receptor; FHCRC, Fred Hutchinson Cancer Research Center; FHVH33, fully human heavy-chain variable domain; Flu, fludarabine; HD, highest dose; ICANS, immune effector cell-associated neurotoxicity syndrome; m, median, mo, months; MSKCC, Memorial Sloan Kettering Cancer Center; NA, not available; NCI, National Cancer Institute; ORR, overall response rate; OS, overall survival; PFS, progression-free survival; PI3K-inh, phosphoinositide 3-kinase inhibitor; scFv, single-chain variable fragment; UPenn, University of Pennsylvania*.

## National Cancer Institute CAR T-Cell

The first-in-human phase 1 clinical trial (NCT02215967) using an anti-BCMA CAR T-cell product with a CD28 costimulatory domain was conducted at the National Cancer Institute (NCI) (21, 44, 67). This phase 1 dose escalation trial analyzed four dose levels: 0.3 × 10^6^, 1 × 10^6^, 3 × 10^6^, and 9 × 10^6^ CAR T-cells/kg of bodyweight. Patients received cyclophosphamide (Cy) and fludarabine (Flu) before BCMA CAR T-cell infusion. Twenty-four heavily treated RRMM patients were enrolled, 16 of them received the highest dose level. The lowest doses showed a minimal anti-MM activity with an overall response rate (ORR) of 20%; however, in the highest dose level the ORR was 81%, 63% of patients achieved a very good partial response (VGPR) or better, and the median event-free survival (EFS) was 31 weeks. Notably, all patients who achieved a partial response (PR) or better were found to be minimal residual disease (MRD)-negative by bone marrow flow cytometry. Better responses were associated with high-peak CAR T-cell levels and with sBCMA decrease. Regarding adverse events (AE), the first two patients treated at the highest dose level developed severe cytokine-release syndrome (CRS), therefore it was decided to limit eligibility to patients with less than 30% of bone marrow infiltration. Nevertheless, CRS was observed in 94% of the patients (grade ≥ 3 in 38%, no CRS-related mortality [CRS-RM]); while neurotoxicity was limited to confusion or delirium in the setting of severe CRS (grade ≥ 3 in 19%) (44, 67). This trial represented the first proof-of-concept that BCMA CAR T-cells are effective against RRMM and is the first description of the loss of BCMA from myeloma cells after BCMA CAR T-cell infusion. However, the high incidence of CRS and the subsequent eligibility restriction to patients with low malignant plasma-cell burden underlined the need for further optimization of this cell product.

## LCAR-B38M (JNJ-68284528)

Nanjing Legend Biotech developed a dual epitope-binding CAR containing two llama-derived heavy chain variable fragments against different BCMA epitopes (VHH1 and VHH2) and a 4-1BB costimulatory domain. The results from the phase 1 clinical trial (LEGEND-2; NCT03090659) have been published in two independent studies and were updated at the last American Society of Hematology (ASH) meeting (45–47). The first study enrolled 57 RRMM patients with a median of 3 prior lines of therapy. All patients received Cy as conditioning regimen and the CAR T-cell dose was given in 3 split infusions (20, 30, and 50% of total dose over 7 days). CRS was seen in 89% of patients (grade ≥ 3 in 7%, no CRS-RM) and grade 1 immune effector cell-associated neurotoxicity syndrome (ICANS) was observed in one patient. The median time to response was 1 month. ORR was observed in 88% of patients (with a CR rate of 74% and MRD-negative disease in 68%). A correlation between BCMA expression and clinical response was not reported. After a median follow-up of 19 months, the median PFS was 20 months in all-treated patients, and 28 months in patients achieving MRD-negativity. The 18-month OS was 68% with a median duration of response (DOR) of 22 months (45).

The second study analyzed 17 RRMM patients who received LCAR-B38M in one vs. three infusions of the total CAR T-cell dose, after lymphodepleting chemotherapy over 3 days. Regarding toxicity, CRS was reported in all patients, 41.2% had grade ≥ 3 and one patient died due to severe CRS and tumor lysis syndrome. Severity of CRS was associated with the amount of BCMA expressed on the clonal plasma cells. There were no differences in terms of response among the two delivery subgroups. The ORR was 88.2%, with 76.5% stringent CR (sCR), and after a median follow-up of 13.9 months, 1-y PFS and 1-y OS were 52.9 and 82.3%, respectively. The investigators found that patients who had received a previous autologous stem cell transplantation (ASCT) had more durable responses, and that the presence of anti-CAR antibodies constituted a high-risk factor for relapse (47).

As a result of these findings, a phase 1b/2 clinical trial is ongoing in the United States (CARTITUDE-1, NCT 03548207), and a phase 2 confirmatory study has started in China (CARTIFAN-1, NCT03758417). The results of 29 patients who received JNJ68284528 in CARTITUDE-1 trial were presented at the last American Society of Clinical Oncology (ASCO) meeting. Patients were extensively pretreated with 5 prior lines of therapy, 86% of the them were triple-refractory (PI, IMiDs, and anti-CD38 antibody) and 27% had high-risk cytogenetic profile. CRS was observed in 93% of patients with one grade 3 event and one grade 5 event, while ICANS occurred in 10% of patients (grade ≥ 3 in 3%). The ORR was 100% with 86% sCR. Of 16 patients in CR who were evaluable for MRD assessment, 13 were MRD neg at 10^−5^ or better and 11 at 10^−6^. Baseline BCMA levels were not predictive of response. After a median follow-up of 11.5 months, the 9-month PFS rate is 86%. The safety and efficacy results are considered largely consistent with LEGEND-2 study (48, 49, 68). In summary, this cell product has shown impressive results in terms of responses with manageable toxicity; nevertheless, at the moment the follow-up of the patients is short. While this fact obviously limits comparability with other cell products, it might also suggest that the efficacy of CAR T-cells is likely to be increased at earlier stages of the disease.

## CART-BCMA

The University of Pennsylvania developed a BCMA CAR with a fully human scFv and 4-1BB costimulatory molecule (69). This CAR was tested in a phase 1 clinical trial (NCT02546167) which included 25 heavily pretreated RRMM patients. This trial analyzed three sequential cohorts: cohort 1, 1 × 10^8^ to 5 × 10^8^ CART-BCMA alone; cohort 2, Cy 1.5 g/m^2^ plus 1 × 10^7^ to 5 × 10^7^ CART-BCMA cells; cohort 3, Cy 1.5 g/m^2^ plus 1 × 10^8^ to 5 × 10^8^ CART-BCMA cells. CAR T-cells were administered in the outpatient setting as split-dose infusions over 3 days (10% on day 0, 30% on day 1, and 60% on day 2). Inclusion into the trial did not require a prespecified level of BCMA expression on MM cells. CRS was observed in 88% of the patients with 32% grade 3 or 4, all of whom were treated at the highest dose of CAR T-cells. Neurotoxicity was seen in 32% and severe neurotoxicity (grade ≥ 3 in 12%) was associated with high tumor burden, high-dose of CAR T-cells and grade 3 or 4 CRS. One patient died after grade 4 CRS complicated by candidemia and disease progression. ORR was 48% (44% in cohort 1, 20% in cohort 2, and 64% in cohort 3). The median PFS (mPFS) was 2.1, 1.9, and 4.2 months for cohorts 1, 2, and 3, respectively, and the median OS (mOS) was 11.8 months, 16.3 months, and not reached for cohorts 1, 2, and 3, respectively. Responses were associated with higher premanufacturing CD4^+^/CD8^+^ T-cell ratio, and frequency of CD45RO^−^CD27^+^CD8^+^ T-cells, suggesting that enrichment of less differentiated, more naïve or stem cell memory-like T-cells is important to improve outcomes of this anti-BCMA CAR T-cell product. No correlation was found between baseline BCMA and expansion or response. In patients responding to the treatment, a decrease in BCMA expression was observed that subsequently increased upon progression (50). This trial provided data that lymphodepletion prior to CAR T-cell therapy is no absolute requirement and the optimal regimen is not clear; however, its administration improved clinical outcomes and short-term CAR T-cell expansion suggesting a favorable impact of conditioning therapy.

## bb2121 (Idecabtagene Vicleucel)

This CAR T-cell construct with 4-1BB as costimulatory molecule was developed by Bluebird bio (70). The multicenter phase 1 study CRB-401 (NCT02658929) consisted of a dose-escalation phase and a dose-expansion phase. The former phase enrolled RRMM patients with ≥50% BCMA expression on plasma cells, while in the latter phase, also patients with less than 50% BCMA expression could be included. Lymphodepletion with Cy and Flu was used before a single infusion of bb2121. In the dose-escalation phase, 21 patients were enrolled and doses of 50 × 10^6^, 150 × 10^6^, 450 × 10^6^, or 800 × 10^6^ CAR T-cells were tested, while in the expansion-phase, 12 patients received 150 × 10^6^ to 450 × 10^6^ CAR T-cells. Regarding safety, 76% of patients developed CRS, thereof 6% grade 3 and no grade 4 or 5; neurologic side effects occurred in 42% of the patients and only one patient (3%) developed a grade 4 ICANS. Respecting efficacy, ORR was 85%, with 45% of patients achieving ≥ CR. At doses ≥150 × 10^6^ (*N* = 30), ORR was 90% with 50% of patients achieving ≥ CR. A total of 16 patients who achieved a response were evaluated for MRD status in the bone marrow. All 16 patients were MRD-negative at a threshold of at least 10^−4^ nucleated cells. Responses appeared independent of sBCMA or tumor BCMA levels. After a median follow-up of 11.3 months, the mPFS in the ≥150 × 10^6^ cohort was 11.8 months. Persistence of bb2121 at 6 and 12 months was detected in 57 and 20% of patients, respectively, and blood CAR T-cell levels were higher in patients who achieved a response (51). Due to these results, a confirmatory single-arm phase II trial (KarMMa; NCT03361748) enrolled 140 patients, of whom 128 patients received the product. The latest results were presented at the last ASCO meeting. Patients were treated at target dose levels of 150–450 x10^6^ CAR T-cells, 84% were triple refractory (PI, IMiD and anti-CD38 antibody) and 35% had high-risk cytogenetic profile. The safety results were consistent with the CRB-401 trial. CRS was observed in 84% of all patients with five, one, and one grade 3, 4, and 5 events, respectively. ICANS occurred in 18% of patients (grade ≥ 3 in 3%). Regarding efficacy results, the ORR was 73% with 33% of patients achieving ≥ CR. Clinically meaningful efficacy was observed across subgroups including high-risk cytogenetics and patients with extramedullary disease. After a median follow-up of 13.3 months, mDOR, mPFS, and mOS were 10.7, 8.8, and 19.4 months, respectively. Efficacy was highest at the target dose of 450 × 10^6^ CAR T-cells (ORR of 82% including 39% CR; mDOR and mPFS of 11.3 and 12.1 months, respectively) (52, 53). In addition, a phase III trial (KarMMa-3; NCT03651128) comparing the efficacy and safety of bb2121 vs. standard triplet regiments in RRMM and (71) clinical trials analyzing the use of this CAR in the second- (KarMMa-2; NCT03601078) or first-line (KarMMa-4; NCT04196491) treatment in high-risk MM patients are underway (72, 73).

## bb21217

Bluebird bio's bb21217 CAR uses the same CAR molecule as bb2121 (70), however, bb21217 is cultured with the phosphoinositide 3-kinase (PI3K) inhibitor bb007 during the manufacturing process, thereby increasing memory-like T-cells (CD62L^+^ and CD27^+^) in order to improve persistence of the CAR T-cells (74, 75). Preliminary results of the phase 1 CRB-402 trial (NCT03274219) were recently presented at ASH. During dose-escalation phase, enrollment was restricted to patients with ≥ 50% BCMA expression on malignant plasma cells. Lymphodepletion protocol was comparable to bb2121 and escalating doses of 150 × 10^6^, 300 × 10^6^, and 450 × 10^6^ CAR T-cells were being tested. Data were presented from 33 heavily pretreated MM patients. CRS and ICANS were observed in 66% of the patients (6% were grade ≥ 3 and one CRS-RM) and 24% of the patients (8% were grade ≥ 3), respectively. The ORR in the first three dose steps were as follows: 83% (≥ CR 33%), 43% (≥ CR 0%), and 57% (≥ CR 14%), respectively. Long-term CAR T-cell persistence was observed in 8/10 evaluable patients at month 6 and 2/2 at month 18. The dose-expansion phase is currently ongoing to further explore the 450 × 10^6^ CAR T-cell dose (54). Initial efficacy results of this next-generation anti-BCMA CAR T-cell are encouraging. The increased proportion of memory-like T-cells has the potential to prolong CAR T-cell persistence and improve potency, however, confirmatory long-term results are still pending.

## JCARH125 (Orvacabtagene Autoleucel)

JCARH125 is an anti-BCMA CAR T-cell product with a fully human scFv and 4-1BB costimulatory domain. It also contains a truncated epidermal growth factor receptor (EGFRt) as a selection marker and safety switch. The construct has shown minimal tonic signaling reducing antigen-independent exhaustion and it is not inhibited by sBCMA. The manufacturing process is optimized for a defined composition of purified CD4^+^ and CD8^+^ CAR T-cells enriched for central memory phenotype cells (76). The phase 1/2 EVOLVE trial (NCT03430011) is currently ongoing. Lymphodepleting chemotherapy consists of Cy and Flu before a single dose of JCARH125 is administered. The first three dose levels evaluated in the dose-escalation phase were 50 and 150 × 10^6^ CAR T-cell. Data on 44 RRMM patients with a median follow-up of 11 weeks was presented. CRS was observed in 80% of patients, CRS grade ≥ 3 in 9%, while neurological events were presented in 25% of patients, grade ≥ 3 in 7%. The ORR in the entire cohort was 82% (≥ CR 27%) (55). Updated results in 62 patients treated with JCARH125 at higher dose levels of 300, 450, and 600 × 10^6^ CAR T-cells were recently presented at ASCO. 41% had high-risk cytogenetic profile and 94% were triple-refractory. CRS was observed in 89%, CRS grade ≥ 3 in 3% and neurotoxicity was seen in 13% and grade ≥ 3 in 3%. Regarding efficacy results, the ORR was 92 and 36% achieving ≥ CR across all dose levels and after a median follow-up of 9.5 months, the mPFS in the 300 × 10^6^ CAR T-cell cohort was 9.3 months (56). Early data reported for this product characterized by a fully human binder, enrichment of central memory cells, and reduced T-cell exhaustion, are promising. It is estimated that recruitment of the phase 2 trial will be completed in 2021.

Currently, there are preliminary data of other ongoing studies, especially in China, which are listed in Table 1. Although data from all these studies are heterogeneous, with differences on patient population, CAR constructs, lymphodepletion protocols, CAR T-cell doses, and grading scales of toxicities, BCMA CAR T-cells showed impressive results achieving deep responses in heavily pretreated MM patients. Nevertheless, there are different caveats regarding the use of CAR T-cells targeting BCMA, for example, the relevance of the heterogeneous BCMA expression on myeloma cells, the impact of sBCMA on the effectiveness of the therapy, and the relapses observed during the follow-up of most patients. Another aspect to highlight is that for most CAR T-cell trials targeting BCMA, the incidence of severe CRS and ICANS is considerably lower as compared to treatment with CD19 CAR T-cells in B-cell malignancies. However, the mechanism of these differences is yet to be elucidated (77, 78).

## CD19

Although widely expressed in B-cell malignancies, CD19 expression in MM is limited. Nevertheless, CD19 CAR T-cells have been effective in a subset of patients. Garfall et al. published the results of a pilot study in which 10 MM patients with early relapse after a first ASCT received a second ASCT followed by CD19 CAR-T cells (CTL019) (79). CD19 expression on myeloma cells by flow cytometry was < 2% in all evaluable patients and negative in 2 subjects. However, an ORR was achieved in 8 patients at 100 days after ASCT (including 1 sCR, 4 VGPR, and 2 PR). Two patients experienced longer PFS after ASCT + CTL019 compared to their previous ASCT, a so called “remission inversion,” suggesting a potential benefit of CD19 CAR T-cell following high-dose melphalan. This might be due to the fact that a significant fraction of myeloma cells expresses CD19 at molecular density which is detectable by direct stochastic optical reconstruction microscopy (dSTORM) but not by flow cytometry (80). Interestingly, less than 100 CD19 molecules are required for myeloma cell detection by CD19 CAR T-cells. In addition, evidence of a less differentiated MM subclone (CD19^+^ CD138^−^) with drug-resistance and disease propagating properties has emerged (81, 82). Despite these encouraging findings, the use of CD19 CAR T-cells as a potential treatment for MM needs to be further explored. Data from an ongoing phase II clinical trial (NCT02794246) of CD19 CAR T-cell as maintenance therapy in RRMM are expected to provide further insights.

Further evidence supporting a role of CD19 CAR T-cells in MM might come from multi-antigen specific CAR T-cell products. Preclinical *ex vivo* experiments showed that myeloma cell depletion was enhanced when CD19 CAR T-cells were tested in combination with BCMA CAR-T cells (79). In a clinical trial, Yan et al. showed that 17 out of 21 patients with RRMM achieved good and durable responses after CD19 and BCMA CAR T-cell infusion (83). Results from the SZ-CART-MM02 study (NCT03455972) presented by Shi et al. during the 45th meeting of European Blood and Marrow Transplantation (EBMT) further supported multi-antigen targeting CAR T-cell therapy in MM. The trial demonstrated that BCMA and CD19 CAR T-cell infusions can be applied as consolidation therapy after tandem ASCT to increase in-depth responses in newly diagnosed (ND) MM patients with high risk features. ORR was 100% with 4 sCR, 3 CR, and 3 VGPR after CAR-T cell infusion. Of four patients in PR after ASCT, 3 achieved VGPR and 1 sCR after CAR T-cell infusion (84). Nevertheless, the additive role of CD19 CAR T-cells needs to be investigated in a randomized study that includes a respective control arm (e.g., BCMA CAR T-cell therapy alone). In this regard, a clinical trial comparing BCMA CAR T-cells alone or in combination with CD19 CAR T-cells is ongoing (NCT03549442).

## Signaling Lymphocytic Activation Family Member 7 (SLAMF7)

SLAMF7 (CS1, CD319, CRACC) is a member of the signaling lymphocytic activation family receptors involved in the regulation of the immune system. SLAMF7 is highly expressed on malignant plasma cells both, in NDMM and RRMM, regardless of the cytogenetic risk stratification (85). A prior study suggests that SLAMF7 has a role in MM cell survival via interaction with the bone marrow stromal niche (86). Intriguingly, pre-malignant stages such as monoclonal gammopathy of undetermined significance and smoldering myeloma retain SLAMF7 expression (85, 87). SLAMF7 is also reported on immune cells, including NK-cells, a subset of T-cells, activated B-cells, dendritic cells and macrophages where it mediates activating or inhibitory functions through intracellular adaptor proteins (88–90). Conversely, hematopoietic stem cells or other solid organ tissues do not express SLAMF7, making it a candidate antigen for immunotherapy. The use of the anti-SLAMF7 antibody elotuzumab in combination with lenalidomide demonstrated efficacy without significant toxicity (91). Therefore, SLAMF7 has been investigated as potential target for CAR T-cell therapy. Our group demonstrated that SLAMF7 CAR T-cells generated from healthy donors and MM patients are able to eradicate either medullary or extramedullary disease in a murine xenograft model (92). Responses were durable and OS was improved. Generation of SLAMF7 CAR T-cells might be challenging due to expression of SLAMF7 on T-lymphocytes. However, it should be noted that SLAMF7 CAR T-cells acquire a SLAMF7-/low phenotype after CAR transduction allowing them to escape from fratricide and to expand to clinically relevant doses (92). Following these results, SLAMF7 CAR T-cell therapy set foot into clinical trials (NCT03710421, NCT04142619, EudraCT Nr.2019-001264-30/CARAMBA-1). The CARAMBA project is a phase 1/2 clinical trial funded from the European Union's Horizon 2020 program and a key innovation of the study relies on the use of a Sleeping Beauty (SB) transposon system as strategy for CAR T-cell engineering. As compared to viral vectors, SB transposition from minicircles vectors provides similar long-lasting transgene expression, superior safety profile and lower manufacturing cost, addressing the challenge of expanding CAR T-cell therapy on global scale (93, 94). To manage undesired toxicity following infusion, this product is equipped with an EGFRt safety switch. In the ongoing NCT04142619 trial, MM patients receive universal “off-the-shelf” SLAMF7 CAR T-cells which contain an inactivation of the TCRα constant (TRAC) gene using transcription activator-like effector nuclease (TALEN) gene-editing technology (95). To prevent self-antigen-driven fratricide, SLAMF7 disruption was included as an additional step during manufacturing.

## CD44v6

CD44v6 is an isoform of the hyaluronate receptor CD44 expressed in MM, acute myeloid leukemia and solid tumors, where it plays a role in tumor growth and dissemination. In MM, CD44v6 is associated with poor prognosis (deletion of 13q14), advanced disease and high-risk features (96). Unlike CD44, the expression of the v6 isoform is relatively tumor-restricted with minimal expression on keratinocytes, activated T-cells and monocytes. Conversely, it is not expressed on hemopoietic stem cells. Casucci et al. reported remarkable antimyeloma effects of CD44v6 CAR T-cells in a mouse xenograft model (97). Only mild and reversible monocytopenia was reported. In this respect, it should be noted that monocyte depletion might have an unexpected benefit since monocytes are the major source of cytokines responsible for CRS and neurotoxicity (98). Bivatuzumab is a mAb tested in a phase 1 clinical trial to direct mertansine activity to CD44v6 expressing solid tumors (99). Although PR has been observed, the trial was discontinued due to severe skin toxicity. Interestingly, Casucci et al. did not observe significant skin-related toxicity after CD44v6 CAR T-cell infusion, potentially because hair follicles represent immune-privileged sites. In addition, CD44v6 CAR T-cell fratricide was not observed. Following these promising results, a first-in-human phase 1/2a clinical trial in RRMM patients treated with CD44v6 CAR T-cells has been registered and it is currently recruiting participants (NCT04097301). The incorporation of a transduction marker (nerve-growth-factor receptor/NGFR) into the transgene allows for CAR T-cell enrichment prior to infusion. Moreover, NGFR can be used to track CAR T-cell *in vivo* to establish expansion and persistence during the follow-up of the patient. To ascertain safety, the HSV-TK Mut2 suicide gene has been included as a safety switch in case of undesired toxicity (100).

## CD138

CD138 is highly expressed in NDMM and RRMM where it promotes tumor growth (101). Although CD138 is broadly expressed in human tissues, CD138 targeting CAR T-cell therapy reportedly favorable toxicity profile but only modest anti-MM efficacy (9, 102). Moreover, immune escape by antigen loss has been reported (103). Therefore, combination of CAR T-cell products against different antigens (CD138 plus BCMA/CD19/CD56/CD38) is currently explored in several clinical trials (NCT03196414, NCT03473496, NCT03271632).

## Natural Killer Group2, Member D (NKG2D) Ligands

Although NKG2D CAR-T cells showed to be effective in a preclinical study against MM cells (104), Baumeister et al. reported no objective response in 5 patients with RRMM treated with first generation NKG2D CAR T-cells (105). This might be explained by the fact that first-generation CAR T-cells have very limited anti-tumor efficacy and persistence. Second, the omission of lymphodepletion therapy may have compromised the engraftment and further limited their expansion. However, no adverse events have been reported. The inclusion of a co-stimulatory domain into the CAR construct and the use of conditioning regimen prior to infusion might scale out NKG2D CAR T-cells efficacy.

## Immunoglobulin Light Chains

B-cell aplasia is a well-known on-target, off-tumor toxicity observed after CD19 CAR T-cell infusion. Targeting antigens that have a more restricted distribution might prevent B-cell depletion. As mature B-cells express either κ or λ light chains, but not both, Ramos et al. showed that κ-directed CAR T-cells might be a promising treatment for B-cell neoplasms, limiting undesired adverse events (10). Although plasma cells generally secrete and do not retain Igs on their surface, myeloma propagating cells expressing surface Igs have been described (106). Therefore, light chains can be a potential CAR target also for MM. In this regard, an anti-κ free light chain mAb was investigated in clinical trials with good responses (107). Unfortunately, κ-directed CAR T-cells did not show discernible responses in 7 patients with κ restricted MM (10).

## CD38

CD38 is highly and uniformly expressed on myeloma cells, making it a candidate target for MM. Several mAbs targeting CD38 demonstrated clinical efficacy and daratumumab has been approved for the treatment of NDMM (108, 109) and RRMM (110–112). Unfortunately, CD38 is also expressed on hematopoietic stem cells, myeloid precursors, and solid tissues including the nervous system (113). Consequently, on-target, off-tumor toxicity might challenge CD38 CAR T-cell therapy. To overcome this issue, several strategies have been proposed. For example, low affinity CD38 CAR T-cells generated through light-chain exchange technology have shown enhanced capacity to discriminate between tumors and CD38 low healthy tissues (114). Also, the incorporation of safety mechanisms into the CAR construct such as caspase-9-based suicide genes (115) or tetracycline inducible CAR design (116) might be a tool to limit off-tumor effects. CD38-targeted CAR T-cells are currently under investigation as a monotherapy for RRMM patients (NCT03464916) or in combination with CD19 (NCT03125577) and BCMA (NCT03767751). Interestingly, a novel antigen receptor structure called dimeric antigen receptor (DAR) targeting CD38 has been designed and delivered into T-cells with a single step non-viral knock-out/knock-in (KOKI) methodology. Preclinical results suggest that DAR-T-cells exhibited a higher cytotoxicity against tumor cells as compared to CAR T-cells (117).

## G Protein-Coupled Receptor Class C Group 5 Member D (GPRC5D)

GPRC5D is widely expressed in MM cells and is associated with poor prognosis (118). Preclinical studies have suggested GPRC5D as promising target for MM (119, 120). Although GPRC5D is expressed in the hair follicle, no skin toxicity was reported in a preclinical model (120). Since GPRC5D expression is independent of BCMA, BCMA/GPRC5D dual-targeted CAR T-cells have recently been demonstrated as a strategy to mitigate BCMA escape-mediated relapse in a xenograft mouse model (121). Consequently, a phase 1 clinical trial of GPRC5D CAR-T cell therapy in patients who had prior BCMA CAR-T cell therapy has been registered (MCARH109).

## CD56

CD56 is highly expressed in myeloma cells but not in normal plasma cells (122). CD56 expression on central and peripheral nervous system entails concerns for neurologic toxicity. In early-phase clinical trial, the anti-CD56 immunotoxin lorvotuzumab mertansine showed modest activity in 37 RRMM patients, but related peripheral neuropathy was observed in about 50% of patients (123). Intriguingly, no serious adverse events have been reported in a case report evaluating CD56 CAR T-cell therapy for rhabdomyosarcoma (124). CD56 CAR T-cells have been shown effective against myeloma cells in a preclinical study (125). Therefore, CAR T-cell therapy targeting CD56 has paved the way for clinical trials in combination with another target antigen, including BCMA (NCT03473496, NCT03271632).

## New York Esophageal Squamous Cell Carcinoma 1 (NY-ESO-1)

The cancer-testis antigen NY-ESO-1 is expressed in a wide variety of malignant neoplasms (126). A study of gene expression profiling conducted on bone marrow biopsies from MM patients revealed that NY-ESO-1 is expressed in 60% of patients at diagnosis and in 100% at relapse, especially in patient with poor prognosis (127, 128). In addition, 10% of MGUS and smoldering MM are NY-ESO-1 positive (128). Conversely, NY-ESO-1 is not expressed on normal tissues, making it a suitable candidate for immunotherapy. As intracellular target, NY-ESO-1 is detected only by TCRs and clinical efficacy of NY-ESO-1 TCR-engineered T-cells in MM patients have been reported (129–131). Interestingly, Schubert et al. managed to make NY-ESO-1 suitable for CAR detection by generating a construct which recognizes the NY-ESO-1/HLA complex on the tumor surface (13). NY-ESO-1 CAR T-cells showed to be effective against myeloma cells *in vitro* (13). A phase 1/2 clinical trial to evaluate safety and effectiveness of NY-ESO-1 CAR T-cells in MM has been registered (NCT03638206). Data from clinical trials of CAR T-cells beyond BCMA are summarized in Table 2.

**Table 2:** Selected non-BCMA CAR T-cell clinical trials in multiple myeloma.

**Registration number**	**Antigen**	**N**	**CAR construct specification**	**Lymphodepletion protocol**	**CAR T-cell dose**	**Response**	**CRS (grade ≥ 3) CRS-related mortality ICANS (grade ≥ 3**
NCT02135406 (79)	CD19	10	Lentivirus 4-1BB scFv NA	HDM + ASCT	1–5 × 10^7^	Response at d100 post-ASCT: ORR 70% (≥ CR 50%)	CRS 10% (0%) CRS-RM 0% ICANS 0%
ChiCTR-OIC-7011272 (83)	CD19 + BCMA	21	Lentivirus 4-1BB scFv murine	Cy/Flu	CD19 CAR T-cell: 1 × 10^6^/kg BCMA CAR T-cell: 1 × 10^6^/kg	ORR 81% (≥ CR 57%)	CRS 86% (5%) CRS-RM 0% ICANS 10% (NA)
NCT03455972 (84)	CD19 + BCMA	10	Lentivirus OX40-CD28 scFv murine	Cy/Bu + ASCT	CD19 CAR T-cell: 1 × 10^7^/kg BCMA CAR T-cell: 1 × 10^7^/kg	Response after ASCT: ORR 100% (≥ CR 70%)	CRS 100% (0%) CRS-RM 0% ICANS 0%
NCT01886976 (9)	CD138	5	Lentivirus 4-1BB scFv NA	Cy VAD PCD	0.756 × 10^7^/kg	ORR 0%	CRS 80% (0%) CRS-RM 0% ICANS 0%
ChiCTR1800018143 (132)	CD38 + BCMA	16	NA 4-1BB scFv NA	Cy/Flu	0.5–4 × 10^6^/kg	ORR 88% (≥ CR 63%)	CRS 63% (25%) CRS-RM 0% ICANS 0%
NCT02203825 (105)	NKG2D ligands	5	Retrovirus scFv human	None	1 × 10^6^-3 × 10^7^	ORR 0%	CRS 0% CRS-RM 0% ICANS 0%
NCT00881920 (10)	κLC	7	Retrovirus CD28 scFv murine	Cy (57%) None (43%)	0.92–1.9 × 10^8^/m^2^	ORR 0%	CRS 0% CRS-RM 0% ICANS 0%
NCT03287804/AUTO2 (133)	APRIL-CAR T-cell against TACI and BCMA	12	Retrovirus CD28 – OX40 scFv	Cy/Flu	15–900 × 10^6^	ORR 43% (≥ CR 0%)	CRS 45% (0%) CRS-RM 0% ICANS 0% (0%)

*APRIL, A Proliferation Inducing Ligand; ASCT, autologous stem cell transplantation; AST, aspartate aminotransferase; Bu, busulfan; BCMA, B-cell maturation antigen; Cy, cyclophosphamide; CR, complete remission; CRS, cytokine-release syndrome; CRS-RM, CRS related-mortality; Flu, fludarabine; HDM, high-dose melphalan; ICANS, immune effector cell-associated neurotoxicity syndrome; κLC, kappa light chain; NA, not available; ORR, overall response rate; PCD, pomalidomide-cyclophosphamide-dexamethasone; scFv, single-chain variable fragment; TACI, transmembrane activator, calcium modulator, and cyclophilin ligand interactor; VAD, vincristine-doxorubicin-dexamethasone*.

## Preclinical Data

Several new targets are currently under investigation for the treatment of MM, including integrin β7 and CD229. Significant efforts have been made to expand the pool of potentially targetable antigens. One of the most interesting strategies relies on the identification of neoantigens generated by post-translational events (such as conformational changes). In this perspective, the activated conformation of integrin β7 has been selected as potential immunotherapeutic target for MM (134). Hosen et al. have developed a mAb (MMG49) that detects an epitope exposed only in the active state of integrin β7. T cells transduced with a MMG49-derived CAR exert anti-MM effects without on-target, off-tumor toxicity (since the inactive state of integrin β7 is not targeted by CAR T-cells) (134). Therefore, a clinical trial to evaluate MMG49 CAR-T cells is expected (134).

CD229 (also known as SLAMF3) is strongly expressed in NDMM and RRMM patients (135). Intriguingly, CD229 is expressed on myeloma-precursor cells responsible for the relapse after treatment. CD229 CAR-T cells have been developed and tested in pre-clinical studies with promising results (136, 137).

## Mechanisms of Resistance to Car T-Cell Therapy in Myeloma and Strategies to Overcome These

**Figure 2:**
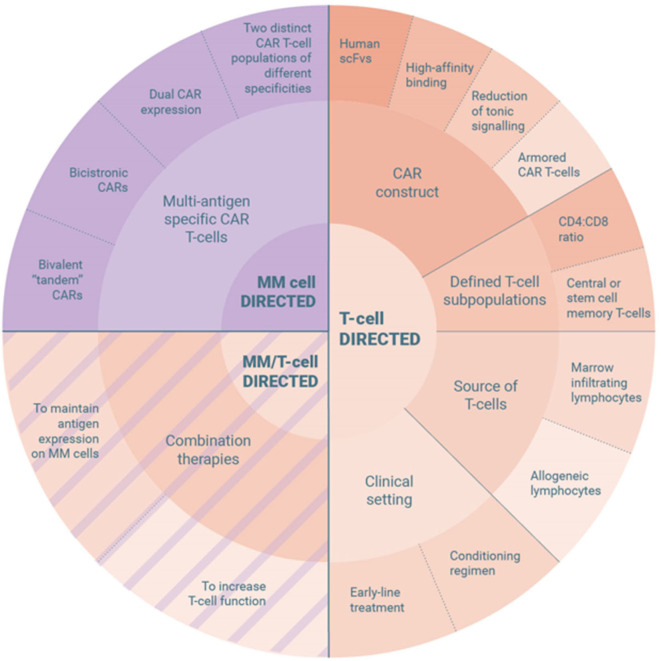
Strategies to overcome resistance mechanisms to CAR T-cell therapy in multiple myeloma.

Despite the recent advances, there are still many questions to be addressed. A meta-analysis of 15 CAR T-cell trials in MM presented at ASH 2019 revealed an ORR of 82%, a pooled relapse rate of 45% and a mPFS of 10 months (138). Reasons why patients relapse or fail to respond include T-cell and tumor cell intrinsic factors. While idecabtagene vicleucel is racing for approval to become the first-in-class CAR T-cell product for treatment of MM, various approaches are tested clinically and preclinically to overcome these mechanisms of resistance and improve clinical efficiency further (Figure 2).

## T-Cell Directed Strategies to Improve Persistency, Potency and Penetration Capacities of Car T-Cells

### Refinement of the CAR Construct

#### Human scFvs

To improve T-cell functionality, one focus is undoubtedly on the refinement of the CAR construct. One milestone toward successful clinical application of CAR T-cells was the recognition that immune rejection responses against the genetically modified T-cells led to reduced persistence and impaired efficacy (139). Ever since, preclinical efforts have been made to reduce the immunogenicity of CAR T-cell by replacing CAR construct components of non-human or partially human nature with fully humanized constructs (140). This has been facilitated by technical advances such as the availability of human B-cell derived scFv phage display libraries. One of the first CAR T-cell products for treatment of MM incorporating a fully human anti-BCMA CAR was developed at MSKCC (76) and has recently been tested clinically as MCARH171 (NCT03070327). Another anti-BCMA CAR T-cell product with a fully human antigen binding domain is the FHVH-BCMA-T containing a heavy-chain-only antigen recognition domain (64, 65). This product is currently under clinical evaluation at the NIH (NCT03602612). Treatment with CT103A (63) (ChiCTR1800018137), a third fully human anti-BCMA CAR T-cell developed by Nanjing IASO Biotherapeutics, was shown to be effective even in patients relapsing after previous CAR T-cell therapy. The elimination of non-human CAR components has thus the potential to break the paradigm to never consider a second dosing of CAR T-cells.

#### High-Affinity Binding/Tuned Affinity

Besides reduced immunogenicity, other characteristics of the targeting moiety are essential for optimal CAR T-cell functionality. These include binding affinity to the antigen, location of the epitope and potentially various other factors (76). In addition to extensive screening analyses of scFv libraries, one approach to generate high-affinity CARs can be the development of dual epitope binding moieties as incorporated in the LCAR-B38M (Legend-2, NCT03090659) (45).

#### Reduction of Tonic Signaling

Another aspect of considerable relevance for the engineering of the CAR construct is the prevention of tonic signaling (141). While low-level TCR tonic signaling represents a physiological process to regulate non-activated T-cells, ligand-independent CAR tonic signaling leads to uncoordinated activation with detrimental effects on CAR functionality. Reasons for increased CAR tonic signaling can be found in the CAR structure. First, the targeting moieties of the CAR often display reduced stability and are therefore at risk of oligomerization or clustering, leading to continuous off-target signaling and development of an exhausted T-cell phenotype (142). While it is yet controversial whether the choice of a defined co-stimulatory domain can reverse CAR tonic signaling (142–144), an attractive option to reduce the likelihood of CAR oligomerization or clustering is the use of alternative targeting moieties, such as centyrinsTM, small monomeric proteins based on a consensus tenascin fibronectin domain (145). One such centyrinTM-based CAR T-cell product is the P-BCMA-101 currently under clinical investigation (NCT03288493) (59, 60). Second, the design of the hinge and spacer domain is crucial for the prevention of tonic signaling, especially when full length IgG derived spacers are incorporated, because those spacers bare the risk of FcγR-mediated interactions (146, 147). Adjusting the hinge and spacer design by replacement of the N-glycosylation site responsible for FcγR-binding has been shown to restore CAR T-cell functionality (147). Albeit their use is largely empirical, most MM trials rely on CAR constructs incorporating CD8α-derived spacers (50, 67, 76) where the problem of optimal immune synapse appears to be less pronounced.

#### Armored CAR T-Cells

Still in its infancy but of considerable clinical potential is the generation of armored CAR T-cells. As in MM the microenvironment can promote immune escape of the tumor cells, armored CAR T-cells are equipped with additional features to overcome the immunosuppressive nature of the MM niche. Examples are CAR T-cells that constitutively express the immune-stimulatory CD40 ligand to prevent antigen loss and induce an endogenous anti-tumor immune response (148) or CAR T-cells that secrete a PD-1 blocking scFv and thus increase anti-tumor efficacy of both, CAR T-cells and tumor-specific bystander T-cells (149). Conceptually attractive, the clinical relevance of such armored CAR T-cells has yet to be demonstrated.

## Selection of Defined T-Cell Subpopulations

While in the first clinical trials, patients received CAR T-cell products comprising random compositions of T-cell subpopulations, evidence has been growing that efficiency and especially persistency can be enhanced through a tailored composition of T-cell subsets (150). Further, it has been demonstrated that enrichment of non-cycling, non-activated early memory CD8^+^ T-cells and CD4^+^ T-cells with early memory features in pre-manufacturing cells is a predictive biomarker of response to BCMA CAR T-cells in MM patients (151). On this basis, various approaches are currently investigated clinically for treatment with BCMA CAR T-cells: For example, the CD4^+^/CD8^+^ ratio is adjusted to 1:1, before gene transfer in the JCARH125 product (NCT03430011) (55) or after gene transfer in the FCARH143 product (NCT03338972) (58), to avoid heterogeneity of the therapeutic agent between patients and to enable enhanced potency through optimized CD4^+^ CAR T-cell help to the CD8^+^ CAR T-cells (150). During generation of bb21217, a follow-on product of bb2121, the PI3K inhibitor bb007 is added to enrich for memory-like T-cells (NCT03274219) (54), because self-signaling by the CAR CD3ζ moiety drives constitutive PI3K activation that favors T-cell terminal differentiation via AKT and mTOR pathways (152). And, although the precise mechanism is still unclear, viral-free generation of the P-BCMA-101 product makes use of the piggyBac® transposon-based DNA modification system that favors the development of a T-stem cell memory phenotype (NCT03288493) (59, 60). However, the eventual clinical benefit of the different strategies to specifically engineer products of a desirable T-cell subset composition remains unclear to date, because follow-up periods are still too limited to assess durability of responses.

## Source of CAR T-Cells

Another approach to obtain T-cells with favorable properties for CAR T-cell generation is the use of bone marrow derived T-cells, termed MILs (marrow-infiltrating lymphocytes) (153). MILs display enhanced memory phenotype, higher CD8^+^/CD4^+^ ratio and better ability to persist long-term (154) and MILs that were genetically modified to express a CAR demonstrated improved anti-myeloma efficacy and reduced phenotypic exhaustion *in vitro*.

The use of allogeneic lymphocytes for the manufacture of CAR T-cells primarily targets the logistical and financial constraints of autologous CAR T-cell therapy, but also represent a source of T-cells with reduced prior exposure to anti-myeloma therapy. The availability of advanced strategies for gene editing, such as CRISPR–Cas9 technology (155) or TALEN-mediated approaches (156), have been key for the required genetic modifications to provide “off-the-shelf” CAR T-cells; such as the elimination of the αβ T-cell receptor to reduce the potential of severe graft-vs.-host disease of allogeneic T-cells or to knock-out the CD52 gene to enable selective lymphodepletion of host lymphocytes by alemtuzumab (157). Different BCMA-directed products have been designed, such as ALLO-715 characterized by TALEN-disrupted TCR-α chain and CD52 loci (158), and P-BCMA-ALLO1 that lacks the endogenous T-cell receptor and β-2 microglobulin (159). The SLAMF7-directed “off-the-shelf” CAR T-cell product termed UCARTCS1 with inactivated TCR-α chain locus and SLAMF7 gene knock-out to reduce fratricide of SLAMF7 positive CAR T-cells (95), is currently investigated clinically in the MELANI-01 trial (NCT04142619). Another promising, protein-based, option to generate TCR-depleted CAR T-cells in a single viral transduction step is the incorporation of a TCR-directed scFv with an endoplasmatic reticulum-retention domain (TCR-KDEL) (160), and clinical testing is on the way. If efficacy and safety of CAR T-cell products generated from healthy donor lymphocytes prove comparable to autologous products, the “off-the-shelf” approach has the potential to facilitate CAR T-cell delivery significantly in the future.

## Clinical Setting of CAR T-Cell Therapy

### Conditioning Therapy

For over a decade, it has been common understanding in the field, that efficacy of adoptive T-cell therapy can be significantly enhanced by prior lymphodepletion. Mechanisms proposed at the time were the elimination of immunosuppressive regulatory T-cells (161) and cellular “sinks” for homeostatic cytokines (162). Recent data suggest that there are further biological effects to the conditioning regimen than the sole lymphodepletion that impact responses to CAR T-cell therapy in Non-Hodgkin lymphoma (163). The beneficial effects of conditioning therapy prior to adoptive T-cell transfer have also been observed in the field of MM (50), even if a variety of regimens with regards to drugs, timing and dosing is being used in the different clinical trials. Studies to elucidate the exact effects of the conditioning treatment are therefore warranted to optimize conditioning regimens further (164).

### CAR T-cell Therapy as Earlier Intervention During MM Disease

MM is associated with adverse changes in the T-cell repertoire (165, 166) and many anti-myeloma therapies are detrimental to the lymphatic cells. A recent study performed at the University of Pennsylvania provides data for the hypothesis that CAR T-cell products generated from leukapheresis samples collected after first line induction therapy would be of greater clinical potential as compared to samples collected from RRMM patients (167). In fact, as safety has been demonstrated for a variety of BCMA CAR T-cell products in phase 1 clinical trials, the evaluation of CAR T-cell therapy at earlier time points during the course of MM disease appears promising. More recent clinical studies like KarMMa-2 (NCT03601078; inclusion of patients with 1 prior line of therapy and high-risk factors), CARTITUDE-2 (NCT04133636; 1-3 prior lines of therapy), and KarMMa-4 (NCT04196491; first-line treatment in high-risk MM patients) are currently putting this hypothesis to the test and results are eagerly awaited.

## Combination Therapy to Improve Efficacy of CAR T-cells

The concept of combination therapies in MM dates back to the last century (168), thus it is not surprising that various efforts are made to identify suitable immunomodulatory drugs for combination with CAR T-cell treatment. Preclinical analyses demonstrated that the addition of lenalidomide during CAR T-cell generation (169) or functional testing (170) enhanced anti-tumor activity of the CAR T-cell product. Besides effects on T-cells and other immune cells, lenalidomide has direct anti-myeloma properties and therefore appears a promising combination partner for CAR T-cell therapy of MM. First trials with BCMA CAR T-cells are evaluating this concept clinically (NCT03070327, NCT04133636).

A clinical observation provided a proof-of-principle for PD-1 blockade as another possible, primarily T-cell directed, combination drug for CAR T-cell therapy (171). In a small case series, one out of five patients who progressed after BCMA CAR T-cell therapy experienced significant, yet transient, expansion of CAR T-cells during pembrolizumab-based salvage treatment. Another instructive clinical observation was reported for a patient who required radiotherapy due to spinal cord compression shortly after BCMA CAR T-cell therapy (172). This patient experienced expansion of his TCR repertoire and durable systemic response not exclusively attributable to radiotherapy, suggesting an abscopal-like synergism between radiotherapy and CAR T-cell treatment.

## Tumor Directed Strategies to Reduce Antigen Loss

Besides adequate cellular functionality, a crucial prerequisite of effective CAR T-cell therapy is the maintenance of relevant antigen expression levels on the tumor surface (173). Interestingly, it is especially in patients with long persistence of CAR T-cells that antigen-negative/low variants have been observed. In clinical trials with CD19 CAR T-cells for treatment of B-cell malignancies, up to 25% of patients with complete remission relapsed with CD19 negative disease (2). This observation is commonly referred to as “antigen loss,” denoting different degrees of antigen reduction caused by different underlying mechanisms. Such mechanisms observed during CD19 CAR T-cell therapy include antigen downregulation, expression of different forms of the antigen lacking the target epitope (termed “antigen escape”) and linage switch to a phenotypically distinct disease (173). As MM is characterized by high clonal competition, the promotion of antigen low/negative clones due to the selective pressure of the CAR T-cells appears a plausible mechanism of resistance. In fact, reduction of BCMA expression levels was reported during BCMA-directed CAR T-cell therapy (50) and development of relapse with both, BCMA positive and negative MM cells was described (44). One approach to mitigate antigen loss is to target more than one antigen.

### Multi-Antigen Specific CAR T-Cell Products

Targeting more than one antigen can be achieved through multispecific CAR T-cell products containing two distinct T-cell populations of different specificities, one T-cell population with dual CAR expression, bicistronic CARs (where two independent CAR molecules are encoded on the same vector), or bivalent “tandem CARs” (recognizing two different antigens) (173), or CARs with three specificities using Designed Ankyrin Repeat Proteins (DARPins) (174). One example for tandem CARs is the APRIL-based CAR targeting both antigens, BCMA and TACI. This resulted in the prevention of a BCMA negative relapse in a preclinical *in vivo* model of MM, whereas CAR T-cells targeting solely BCMA did not (175). The APRIL-based CAR T-cell product is currently investigated in the AUTO2 trial (NCT03287804) (133). As mentioned previously, other multispecific CAR T-cell products containing a bicistronic BCMA and SLAMF7-directed CAR (176, 177) or simultaneously manufactured BCMA- and GPRC5D-directed CAR T-cells (121) have been investigated preclinically, and clinical testing of bispecific CAR T-cells targeting BCMA and CD38 (132) or BCMA and CD19 (178) are ongoing in China. Even if preliminary data of treatment with bispecific CAR T-cells has been encouraging, the comparison of bispecific and single-antigen specific CAR T-cells needs to be performed in randomized clinical trials (132). While targeting more than one antigen has the potential to increase CAR T-cell efficacy and reduce the risk of antigen loss, it increases the risk of on-target, off-tumor toxicities and the incorporation of respective safety measures is highly recommended.

### Modulation of Antigen Expression on Myeloma Cells

Another strategy to reduce antigen loss is to improve maintenance of the antigen expression on MM cells. This can be achieved by pharmacological induction of increased surface antigen density, as demonstrated for the CD38-modulators all-trans retinoid acid (179), panobinostat (180), and ricolinostat (181). Furthermore, the identification of γ-secretase mediated cleavage of BCMA enabling the release of soluble BCMA has led to preclinical evaluation of the γ-secretase inhibitors RO4929097 and LY3039478 to increase BCMA expression on MM cells (182). Both agents conferred enhanced CAR T-cell reactivity to MM cells correlating with increased BCMA surface expression and reduced shedding of soluble BCMA. Interestingly, only three oral doses of LY3039478 increased the percentage of BCMA positive MM cells from below 30 to 99.3% in a small cohort of patients. These observations prompted the initiation of a clinical trial investigating the combination of BCMA CAR T-cells with pharmacological γ-secretase inhibition (183). First results are promising, especially as responses were observed in patients relapsing from previous BCMA-directed therapy.

## CAR T-Cell Related Toxicities

The use of this novel therapy has brought in clinical practice—in addition to the expected chemotherapy-related toxicities—the development of new specific and challenging side effects, including: CRS, neurological toxicity [also referred to as CAR-related encephalopathy syndrome (CRES)] also known as ICANS, on-target, off-tumor toxicity, anaphylaxis/allergy, insertional oncogenesis, tumor lysis syndrome, and delayed toxicities such as infections and prolonged cytopenia (184–186). An extensive review of CAR T-cell related toxicities is not the scope of this manuscript, as different publications have addressed this topic in detail (77, 184, 185, 187–189). Therefore, only a short overview will be provided.

CRS is the most frequent serious acute CAR T-cell related toxicity, with an absolute incidence of 30–100%, and 10–30% for CRS grade ≥ 3 (78). It is triggered by the activation and expansion of the CAR T-cells and lysis of normal and tumor cells, leading to the release of effector cytokines such as IL-2, tumor necrosis factor-α (TNF-α) and interferon-γ (IFN-γ) (190). Subsequently, the monocyte—macrophage system is activated and distinct pro-inflammatory cytokines such as IL-1, IL-6, IL-10, IFN-γ, monocyte chemoattractant protein-1 (MCP-1), and inducible nitric oxide synthase (iNOS) are released, leading to increased levels of C-reactive protein and ferritin (191–193). The clinical presentation can range from flu-like symptoms to life-threatening manifestations (hypotension, capillary leak, coagulopathy, and organ dysfunction). The management of CRS requires a pro*per severity* grading (185, 186, 194, 195). The treatment of CRS is based on supportive care with antipyretics, IV hydration, vasopressor support, supplemental oxygen and, in severe cases, the use of the specific IL-6 receptor antagonist tocilizumab and corticosteroids (185, 189, 194, 196, 197). Another potential, but currently still experimental procedure to prevent CRS could be the use of dasatinib that acts as a pharmacologic on/off switch for CAR T-cells (198).

ICANS is the second most frequent adverse event after CAR T-cell infusion, with an incidence of 0–87% (2–4, 187). The pathophysiology is not completely understood; nevertheless, there is evidence pointing toward an impairment of the blood brain barrier due to increased levels of TNF-α, IL-6, IL-1, IFN-γ, IL-10, IL-8, MCP-1, quinolinic acid, and glutamate (199–201). ICANS can develop concurrently with or without CRS (199). Symptoms are variable, ranging from toxic encephalopathy with word-finding difficulty, aphasia, and confusion to more severe cases with coma, seizures and cerebral edema (185, 186, 189). The treatment is based on supportive care and corticosteroids are reserved for grade 3 or 4 ICANS. The use of tocilizumab is recommended only if there is concurrent CRS (185, 188, 189, 199, 202).

Regarding on-target, off-tumor toxicity, the typical example is the development of B-cell aplasia and hypogammaglobulinemia following treatment with CD19 CAR T-cells (185, 188, 189). Further examples of on-target, off-tumor toxicity include carboxyanhydrase-IX-specific CAR T-cell therapy for renal cell carcinoma that resulted in the development of cholestasis due to expression of this antigen on bile duct epithelium (203), and unfortunately, the infusion of HER-2/neu CAR T-cells in a patient with advanced colonic cancer that led to respiratory distress and cardiac arrest which was attributed to putative low levels of HER-2/neu expression on lung epithelial cells (204).

Another theoretical danger with the use of genetically modified cell products is the possibility of insertional oncogenesis; nevertheless, no cases have been reported so far (205, 206). However, in the case of a patient with chronic lymphocytic leukemia receiving CD19 CAR T-cells, a clonal expansion of a single CAR T-cell with biallelic *TET2* dysfunction was reported (207). Therefore, strict long-term monitoring of patients receiving CAR T-cell therapy should be implemented.

## Clinical and Logistical Challenges of Incorporating CAR T-Cells into the Current Treatment Landscape in MM

The optimal timing of CAR T-cell therapy in the sequence of anti-myeloma therapy is still an open question. The most urgent unmet clinical need remains in patient subgroups with extensive pretreatment history who are often multi-agent refractory and suffer from highly aggressive disease. However, it is postulated that CAR T-cell products generated in the RR setting display inferior clinical efficacy than products generated from patients after first-line induction therapy (167). In fact, a recent meta-analysis of 15 clinical trials (thereof 14 in RRMM, only 1 in NDMM) documented a CR rate of 36% with MRD negativity in 77% (208) and a mPFS of 10 months. Therefore, the curative potential in the respective patient cohorts is limited to date. The implementation of a CAR T-cell therapy as early intervention appears more promising, particularly since it is designed as a single dose therapy. However, the last decade has brought in place a variety of highly effective anti-MM drugs that are increasingly moving to frontline therapy and improve survival (209–211). Therefore, deliberate study designs and long follow-up periods will be required for head-to-head comparisons of single dose CAR T-cell treatment and continuous “conventional” anti-myeloma therapy.

However, such data will be essential for the fate of CAR T-cell therapy in MM, especially when socioeconomic considerations are taken into account. The generation of autologous CAR T-cell products is a time consuming, labor- and resource-intensive procedure and special prerequisites are required for transportation, storage and administration. In this regard, efforts to generate “off-the-shelf” CAR T-cells as well as standardized logistics at certified medical facilities are essential developments. However, for broad application of CAR T-cells, a favorable cost-effectiveness ratio will be required. Treatment costs are expected on a similar level as the FDA/EMA approved anti-CD19 CAR T-cell products ($475.000, Kymriah® $373.000, Yescarta®). For both products, the Institute for Clinical and Economic Research (ICER) estimated a cost-effectiveness below $ 150.000 per quality-adjusted life-year (QALY) gained (212), a threshold that is commonly considered cost effective. However, the long-term effectiveness is the critical, yet unknown determinant of a product's cost-effectiveness (213). Therefore, incorporation into a curative strategy will be decisive for CAR T-cells as anti-myeloma treatment in the future.

## Conclusions

The treatment arsenal in MM has grown considerably during the last decade and idecabtagene vicleucel and other CAR T-cell products are on their way to approval. However, the clinical value of CAR T-cells in the current landscape of myeloma therapies remains to be determined. Besides the various advanced T-cell engineering strategies, clinical, and economic factors will have to be taken into account for successful incorporation of CAR T-cells into existing treatment paradigms. Optimal patient selection will be crucial, not only with regards to timing in the course of the disease, but also concerning disease-specific factors like extramedullary manifestations or cytogenetic high-risk features. Also, feasibility of the manufacturing in terms of production capacities and time interval from leukapheresis to adoptive transfer will impact whether CAR T-cells take root into clinical routine. And although no such cell product is currently commercially available for the treatment of MM, treatment costs are expected on a comparable scale as the approved CD19 CAR T-cell products. Eventually, more robust long-term data and, ideally, evidence of curative potential will shape the future of CAR T-cells in this disease. Therefore, clinicians, researchers and society need to collaborate to maximize patients' benefit.

## Author Contributions

LR-L, MG, and SD wrote and reviewed the manuscript. CF, MH, and HE reviewed the manuscript. All authors approved the final version of the manuscript.

## Conflict of Interest

The authors declare that the research was conducted in the absence of any commercial or financial relationships that could be construed as a potential conflict of interest.
